# Successful Integration of Community-Based Rapid Antigen Testing for COVID-19 and Malaria in Mali

**DOI:** 10.4269/ajtmh.23-0855

**Published:** 2025-03-05

**Authors:** Guillaume Breton, Issouf Maïga, Aboubacar Maïga, Gabrièle Laborde Balen, Luis Sagaon-Teyssier, Anne Hoppe, Odé Kanku Kabemba, Boubacar Cissé, Fatou Diawara Traore

**Affiliations:** ^1^ SOLTHIS, Paris, France;; ^2^ Department of Infectious Diseases, Pitié-Salpêtrière Hospital, Paris, France;; ^3^ Solthis, Bamako, Mali;; ^4^ IRD-TransVIHMI UMI 233/ INSERM 1175 Université de Montpellier, Montpellier, France;; ^5^ Aix Marseille Univ, Inserm, IRD, SESSTIM, Sciences Economiques & Sociales de la Santé & Traitement de l’Information Médicale, ISSPAM, Marseille, France;; ^6^ FIND, Geneva, Switzerland;; ^7^ Elizabeth Glaser Pediatric AIDS Foundation, Geneva, Switzerland;; ^8^ National Institut of Public Health (INSP), Bamako, Mali

## Abstract

In Mali, access to health care facilities (HCFs) is limited due to distance and transportation costs, and this limitation may have led to an under detection of COVID-19 cases. This prospective randomized study compared a community-based, integrated COVID-19 and malaria testing strategy (intervention arm) to the national standard-of-care strategy (SOC arm). Four health areas were randomly assigned. All people seeking care who accepted the study were enrolled by community health workers (CHWs) and screened for COVID-19 symptoms. In the intervention arm, CHWs performed COVID-19 and/or malaria antigen rapid diagnostic tests (Ag-RDTs) for patients who met clinical criteria for possible COVID-19, including fever. In the SOC arm, CHWs referred patients who met clinical criteria for possible COVID-19, including fever, to the nearest health care facility (HCF) where COVID-19 and/or malaria Ag-RDTs were performed. Febrile patients refusing referral were tested for malaria by CHWs. Among 1,164 patients enrolled, 73% had fever and 72% meet clinical criteria for possible COVID-19. Malaria Ag-RDTs were performed in 79% and 3% COVID-19 suspected patients in intervention and SOC arms, respectively (*P* <0.001). Only three patients tested positive for COVID-19. Among 449 patients referred to HCFs, 248 refused to go to the HCFs, and only 10 of 201 who agreed to the referral actually reached one. Among febrile patients, 75% and 34% received malaria treatment in intervention and SOC arms, respectively (*P* <0.001). Integration of community-based testing for COVID-19 and malaria Ag-RDTs was found to be feasible. However, limited access to HCFs in rural areas highlights the need for treatment services to be available at the community level.

## INTRODUCTION

In March 2020, the World Health Organization (WHO) declared the Coronavirus disease 2019 (COVID-19) outbreak, which originated in Wuhan, China in November 2019, to be a global pandemic. By the end of 2019, over 722 million cases had been confirmed, and nearly 6,981,000 deaths had occurred worldwide.^1^ To minimize transmission, availability of diagnostic tools such as easy-to-use antigen tests (Ag-RDTs) at or near the point of care, without requiring costly laboratory or specialized equipment, and expanded access to testing, particularly in low-income countries is an essential part of an effective response to COVID-19, enabling early identification and isolation of cases.^2^ Consequently, the WHO recommended using Ag-RDTs in various settings where nucleic acid amplification tests are not available or where long result wait times would undermine the clinical benefits of screening.^3^

Mali appears to have been relatively less impacted by the global pandemic. As of January 31, 2022, 36,046 cases and 715 deaths were reported.^1^ However, the WHO estimates that only 14.2% of COVID-19 infections were actually detected in Africa^4^ and a seroprevalence study in Mali revealed a significant underestimation of cases,^5^ likely due to the high prevalence of asymptomatic or pauci-symptomatic cases in a predominantly young population, and limited testing capacity which relied mainly on polymerase chain reaction (PCR) testing.^6^ Notably, PCR was only available in Bamako, the capital, and testing of symptomatic patients in healthcare facilities (HCFs) was insufficient, with over 80% of the tests administered to travelers. The ECoVAM study, conducted from October 2021 to January 2022, evaluated a triage and testing strategy based on clinical criteria and Ag-RDTs for COVID-19 Ag in seven HCFs, in Mali.^7^ In this ECoVAM study, 58% (1,405/2,347) patients seeking care and enrolled had clinical symptoms suggestive of COVID-19, of which 25% tested positive by Ag-RDT, highlighting the wide utility of Ag-RDTs in detecting COVID-19.^7^ However, due to the influx of patients and the workload of healthcare workers, participation in the study could only be offered to 38% of eligible patients. In addition, we observed that caregivers had difficulty applying the WHO COVID-19 criteria, with nearly 15% of classification errors, mainly linked to misunderstanding of symptom duration and errors in counting the number of minor criteria.

In Mali, more than half of the population lives far from HCFs. To meet the primary healthcare needs of the population residing more than 5km from the Community Health Centre (CHC) – the most decentralised HCF in the health pyramid – Mali deploys community health workers (CHWs), supported by community relays. The CHWs are members of the community they serve, have basic training, and are responsible for providing primary care, prevention (maternal and child health, vaccination, and malaria control) and community-based epidemiological surveillance, as well as for referring patients to the CHC, when needed. Although CHW activities focus mainly on maternal and child health, the integration of the care of adults into their services has been implemented since 2017. For example, CHWs play a critical role in community-based malaria screening using Ag-RDTs and in treating of non-severe cases.

In the response to the COVID-19 pandemic, CHWs were tasked with raising public awareness, assisting in identifying suspected COVID-19 index and contact cases, and facilitating their referral to CHCs. With the availability of COVID-19 Ag-RDTs, integrating COVID-19 screening into CHW activities became feasible, allowing for immediate confirmation of COVID-19 cases and efficient contact tracing, thereby improving equity in healthcare access and reducing the workload of healthcare workers in HCFs. In the present study, we assess the feasibility, acceptability, and cost-effectiveness of an integrated community-based screening strategy using COVID-19 and malaria Ag-RDTs for individuals with clinical suspicion of COVID-19 and/or fever, as well as for COVID-19 contacts.

## MATERIALS AND METHODS

### Study design.

This was a prospective interventional study comparing a systematic community-based COVID-19 and malaria triage and testing strategy using Ag-RDTs for suspected COVID-19 cases and contacts to the national standard-of-care (SOC) strategy.

### Setting and population.

The study took place in the Fana district, Mali, within four health areas (Marakacoungo, Tingolé, Koni, and Fana Central) with an estimated population of 26,433 inhabitants (11,683 adults) residing more than 5km from the nearest CHC. These areas were served by 25 CHWs and 38 community relays. Health areas were randomly assigned to intervention arm (Marakacoungo and Tingolé) and SOC arm (Koni and Fana Central). The CHWs and community relays identified sick adults during consultations at health huts or routine home visits, and they invited them to participate, regardless of symptoms.

#### Inclusion criteria.

Aged ≥18 years; symptomatic patients or household contacts of confirmed COVID-19 patients.

#### Exclusion criteria.

Health problems related to surgical or obstetrical pathologies; Pre- or post-natal follow-up; Life-threatening emergencies; Inability to provide informed consent due to physical or mental health conditions.

### Study procedures.

The CHWs were trained in the use of SARS-CoV-2 Ag-RDTs (STANDARD Q COVID-19 Ag Test, SD Biosensor) with nasopharyngeal swabs according to manufacturer’s instructions, including personal protective equipment for sampling (surgical mask, gloves, gown, and glasses).

Awareness campaigns were launched at the beginning of the study, with meetings held with heads of families and women in each village, followed by broader village gathering. Additionally, radio messages promoting the study were broadcasted on local radio stations throughout the study period.

After signing informed consent, the recruited patients underwent a clinical evaluation of COVID-19 symptoms based on WHO criteria^8^ and a temperature measurement. The Kobo tool box application^9^ was used to assist CHWs in classification. All patients meeting criteria for suspected COVID-19 were classified as “suspected COVID-19 patients”.

#### Intervention arm.

All suspected COVID-19 patients were offered nasopharyngeal swab and COVID-19 Ag-RDTs administered by CHWs at home or at a health hut. Contact cases were offered Ag-RDTs regardless of symptoms. Patients testing positive or undetermined on Ag-RDT were referred to a CHC.

#### SOC arm.

In line with the national standard of care strategy, all suspected COVID-19 patients were referred to a CHC, where laboratory technicians conducted nasopharyngeal swabs and COVID-19 Ag-RDTs. Results for positive tests were transmitted by phone to CHWs, who were responsible for contact tracing, and facilitating referrals to CHCs.

At CHCs, COVID-19 cases were managed according to national recommendations, with severity evaluation, home care for mild cases, and transfers by oxygen-equipped ambulance to the CSREF in Fana or the Mali Hospital for the most serious cases.

In both study arms, following national malaria treatment guidelines, CHWs performed malaria Ag-RDTs on all patients with fever who were not referred to CHCs, while laboratory technicians performed malaria Ag-RDTs for patients accessing CHCs. Malaria cases identified by CHWs or at the CHCs were treated with Artemisinin-based combination therapy.

### Data collection and supervision.

The following data were collected: demographic information, comorbidities, fever or subjective fever, criteria of suspected COVID-19, severity indicators, referrals to CHCs, reasons for refusals, medical treatment and clinical outcome at day 10 for COVID-19 cases. In the intervention arm, CHWs documented sample collection, Ag-RDTs results, and reason for non-collection or non-testing. Picture of the Ag-RDTs was taken and uploaded on Kobo toolbox application for retrospective quality control by the study team.

Supervision visits were conducted by the study team and the CHC directors. Due to security concerns following a terrorist attack on the Fana police station, field visits were interrupted during July and replaced by remote supervision by phone.

## STATISTICAL ANALYSES

The study was powered to detect differences in the total number of COVID-19 cases identified through Ag-RDTs between the intervention arm and the SOC arm. The incidence of suspected COVID-19 cases in adults (150/100,000) and the incidence of COVID-19 (35/100,000) were drawn on ECoVAM study results. We hypothesized that the proportion of COVID-19 suspect cases tested with Ag-RDTs among the population would be 56% in the intervention arm and 19% in the SOC arm. With a power of 90%, and an alpha risk of 5%, the required size of adult population residing in health areas was 2,322 for each arm. Given the unpredictable variability in the incidence of COVID-19 and assumptions made regarding strategy effectiveness, health areas with population twice as large as calculated (5,879 adults in intervention arm and 5,804 adults in SOC arm) were selected to ensure study objectives were met.

Following outcomes, including the proportion of suspected COVID-19 patients, those who received COVID-19 Ag-RDTs, febrile patients tested with malaria Ag-RDTs, and patients treated for malaria were estimated with their two-sided 95% confidence intervals. Comparisons between arms in univariate analysis were performed using Chi-squared or Fisher’s exact tests.

### Socio-anthropological component.

Semi-structured interviews were conducted face-to-face to assess perceptions of COVID-19 and the acceptability of the community-based COVID-19 Ag-RDT screening strategy among suspected COVID-19 patients, contacts, CHWs, CHC directors in charge of CHWs supervision, and community leaders. Interviews were recorded, manually transcribed and analyzed thematically, focusing on perceptions of COVID-19, the integration of the management of adults into CHWs activities, triage strategy, and the acceptability of Ag-RDTs and referrals.

### Cost-effectiveness component.

An economic evaluation was conducted to determine the cost of implementing a community-based COVID-19 testing strategy by CHWs using Ag-RDTs. Healthcare resources included: awareness activities; Ag-RDTs for malaria and COVID-19; materials for hygiene and waste management; individual protective equipment; CHWs training for COVID-19 Ag-RDT; and human resources involved in the diagnostic activities. In the economic calculations, we included all healthcare resources that would be part of routine activities. Research-related costs were excluded from the economic evaluation.

Effectiveness was measured by the number of individuals tested for COVID-19 with Ag-RDT during the study, representing each strategy’s impact on COVID-19 testing access. The effectiveness was presented as the number of Ag-RDTs per 1,000 patients enrolled per study for each arm (${\bar{E}}_{\text{arm}}$). The net cost of the intervention was calculated as:

Net Cost = Cost Intervention – Cost Standard of Care

The incremental cost-effectiveness ratio (ICER) was calculated as:

ICER = Net Cost/Δ Ag-RDTs per 1,000 Patients reached by CHWs

$$\text{with}\;\text{Δ}\_\text{tested}\;\text{Cases}=\bar{E}\;\text{intervention}-\bar{E}\;\text{standard}\;\text{of}\;\text{Care}$$

### Ethics.

The study was approved by INSP Ethics Committee (N° 07/2022/CE_INSP). All participants provided written informed consent. For illiterate persons, consent was taken in the presence of a witness and the consent was signed by fingerprinting.

## RESULTS

### Population characteristic.

Between June 2022 and September 2022, during the malaria transmission season, 2,555 sick adults consulted a CHW and were offered study participation. Of the 2,555 offered participation, 1,164 (46%) were included – 778 in the intervention arm and 386 in the SOC arm. Inclusion refusal rates were higher in the SOC arm (62% versus 49%) (*P* <0.0001) without male or female differences. The main reasons for refusal in the intervention arm were reluctance to provide signed informed consent (57%) and belief that COVID-19 does not exist (12%). This was more common among men (18%) than women (9%) (*P* <0.01). In the SOC arm, the most cited refusal reasons were disbelief in COVID-19 (31%) and reluctance to provide signed informed consent (14%). Refusal by the household head, usually a man, restricted participation more for women than men (15% versus 9%, *P* = 0.04).

Overall, more women (695/1,164; 60%) than men (469/1,164; 40%) participated. Points of care access included: health hut visits (617/1,164, 53%), routine home visit by CHWs (268/1,164, 23%), targeted home visit by CHWs after a phone call from a community relay (279/1,164, 24%). The median age was 30 years (IQR 24–42). The proportion of people over 50 years of age was 20% (233/1164). The frequency of comorbidities (mainly hypertension) was 11% (128/1,164). The median household size was 10 (IQR 6–16).

### Clinical presentation.

Among the 1,164 patients included, 845 (73%) had fever and 835 (72%) met criteria for suspected COVID-19. Among 835 suspected COVID-19 patients, 752 (90%) had fever, 482 (59%) had major criteria, and 353 (41%) had minor criteria for COVID-19.

### COVID-19 RDTs uptake.

**Figure 1.**
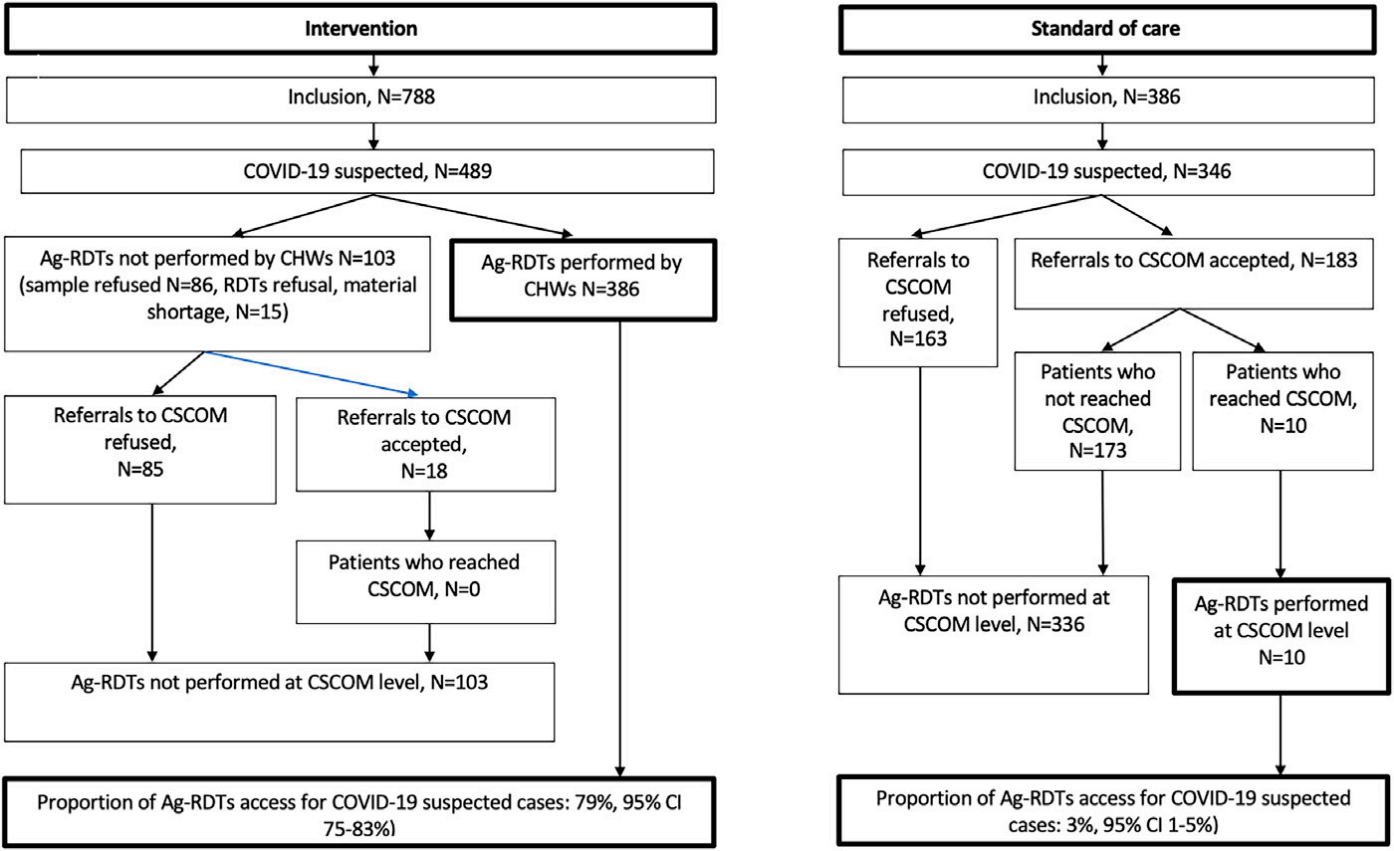
COVID-19 screening and access to COVID-19 Ag-RDTs.

In the intervention arm, 388 out of the 489 (79%) COVID-19 suspected patients received a nasopharyngeal swab. Nasopharyngeal swab was not performed in 15 cases due to supply shortage and in 86 cases due to patient refusal, with refusal rate higher among men than women (23% versus 14%, *P* = 0.008). Ag-RDTs were performed in 386/489 (79%) COVID-19 suspected patients, with 2 patients testing positive (0.5%) (Figure 1).

**Figure 2.**
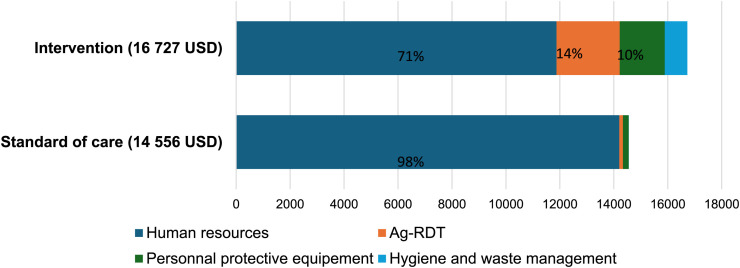
Total cost of strategies.

In the SOC arm, 183 of the 346 (53%) COVID-19 suspected patients accepted referral to the CHC. Among the 163 refusals, the reasons given by 142 patients were: transport costs (49%), time constraints (45%), disbelief in COVID-19 (4%), other (3%), with no male/female differences. Patients referred by female CHWs were less likely to refuse than those referred by male CHWs (33% vs 57%, *P* <0.001) regardless of patient sex. Of the 183 patients who accepted referral, 10 visited the CHC, all of whom received an Ag-RDTs, with 1 testing positive (Figure 2).

Overall, uptake of COVID-19 Ag-RDTs was significantly higher in the intervention arm (386/489, 79%, 95% CI 75–83%) compared to the SOC arm (10/346, 3%, 95% CI 1–5%), *P* <0.001. The detection rate of COVID-19 among suspected cases was similar between the two arms (2/489 in the intervention and 1/346 in the SOC arm 1/346).

Contact case tracing and testing was not evaluated due to the low number of COVID-19 cases.

### Access to HCF for COVID-19 testing.

Overall, access to HCFs after a referral by CHWs among patients meeting criteria for suspected COVID-19 was 10/449 (2%). This result was observed irrespective of the reason for referral and the study arm: none among 103 patients meeting criteria for COVID-19, the intervention arm who refused tests by HCWs (*n* = 86), or for who test was not available (*n* = 15.) and 10 among 346 patients meeting criteria for COVID-19 in the SOC arm.

### Quality control of COVID-19 test interpretation by CHWs.

Of the 386 Ag-RDTs performed by CHWs in the intervention arm, 386 pictures were uploaded in the kobo toolbox application. The interpretation was correct for 384 (99.5%). Two invalid tests were classified as negative.

### Malaria Ag-RDTs uptake in febrile patients.

In the intervention arm, 482 of 495 febrile patients (97%) received a malaria Ag-RDT which was positive in 372 cases (77%). All patients with positive results were treated: 372/372 (100%). In all, 372 of 495 febrile patients (75%, 95% CI 71–79%) received malaria treatment.

In the SOC arm, among 350 febrile patients, 175 patients who were also suspected of having COVID-19 agreed to be referred to the CHC and therefore did not receive malaria testing or treatment by CHWs. Ultimately, 10 of 175 reached the CHC, where they then received malaria Ag-RDTs, and 7/10 received malaria treatment.

In contrast, among 155 febrile patients who were also suspected of having COVID-19 but refused referral, as well as the 20 febrile patients without COVID-19 suspicion, 157 of 175 (90%) received care provided by CHWs: 113 of 157 (72%) tested positive for malaria and subsequently received malaria treatment.

Overall, access to malaria treatment was lower in the SOC arm (120/350; 34% 95%CI 29–39%) than in the intervention arm (372/495; 75% 95% CI 71–79%) *P* <0.0001. The lower access to malaria treatment in the SOC arm is due to the fact that CHWs did not offer malaria Ag-RDTs to febrile patients suspected of COVID-19 who agreed to be referred to the CHC, where this test would have been performed had they attended.

### Socio-anthropological component.

Semi-structured interviews were conducted in the four areas in September 2022, among 28 suspected COVID-19 patients (10 men, 18 women, mean age: 37 years), 25 CHWs (7 men, 18 women, mean age: 27 years), 11 community relays (9 men, 2 women, median age: 42 years), 12 community leaders (7 men, 5 women, mean age: 62 years), and 4 CHCs directors (4 men, mean age: 41 years).

### Patients’ perception of the strategies.

#### Intervention arm.

Key reasons for acceptance included the perception of COVID-19 as a serious disease, a pandemic affecting Mali; endorsement by village authorities; trust in the Community Health Workers (CHWs); difficulty of refusing screening during consultation; and advantages of the test itself – it was free, performed on-site, and provided immediate results. Reasons for refusal were linked to prior negative experiences with past projects in some villages, where promised financial aid was distributed to only a portion of the population, creating distrust toward any “COVID-19 project.” Additional factors included disbelief in the disease due to circulating misinformation and lack of direct or indirect experience with COVID-19, fear of nasal swabbing, stigma from family or friends in the event of a positive test, prioritizing the treatment of the condition motivating the consultation (usually malaria), and, for women, dependence on the family head, who is the primary decision-maker regarding health matters.

#### SOC arm.

Reasons for accepting or refusing screening were generally similar to those in intervention areas. However, only a small proportion of those who accepted the protocol actually went to the CHC for testing. Reasons included the overlap of the study period with fieldwork and the risk of losing a day’s wages, the distance from home to the CHC, challenges accessing the center during the rainy season (e.g., flooded roads), high travel costs, and the perception of COVID-19 as a mild illness (comparable to a common cold), making the benefit of referral seem low. The few individuals who did go to the CHC were those with deteriorated health. The CHC was thus viewed as a last resort, with minor illnesses managed at the community level.

### CHWs’ perception of the strategy.

#### Intervention arm.

Performing COVID-19 Ag-RDTs was viewed by the CHWs as the acquisition of a new skill, which was gratifying as it represented a “medical gesture.” Extending their activities to a male adult audience increased recognition of their social role beyond their usual work focused on maternal and child health. The project strengthened their relationship with the CHC directors and with community relays. CHWs noted the strategy’s effectiveness in promoting screening acceptance. Additionally, the allowances received improved their living conditions, as their salaries were often paid irregularly.

#### SOC arm.

CHWs regretted not being able to perform COVID-19 Ag-RDTs, which could have enhanced their role within the community. They also expressed disappointment at the ineffectiveness of referrals, as well as missed opportunities for testing.

### Perceptions of the strategy by the community and CHC’s directors.

The Community Relay and Community Leaders supported the intervention strategy, as it brought care closer to the community, avoiding travel and costs. They all expressed confidence in “their” CHW and were in favor of expanding their roles and skills. CHC’s directors also supported this strategy. Some initially had reservations about non-medical personnel performing a technical procedure, but supervisions reassured them, and they recognized the number and quality of screenings conducted. In health areas implementing SOC arm, the low attendance of referred patients reinforced the belief that this strategy was ineffective. Opinions among directors on whether CHWs should deliver COVID-19 treatment were divided: some felt that treatment should remain within CHC responsibility; others saw it as an essential complement to the intervention strategy.

### Perception of malaria testing.

CHWs have used Ag-RDTs for years to screen and treat malaria in pregnant women and children. Since 2020, screening has been extended to male dults. The Ag-RDT is free, and malaria treatment costs no more than 1,000 FCFA (1.7 USD). Extending malaria screening to all adults was viewed very positively by the population. Malaria is considered a familiar disease, “a local issue.” The free test, immediate results, low treatment cost, and on-site care from known personnel were universally praised. CHWs and CHC’s directors also had a very positive perception of this practice, which improves healthcare access and reduces mortality. Consultations for malaria symptoms were seen as an opportunity to propose a COVID-19 test, increasing COVID-19 screenings, as patients would not have sought care spontaneously without suspected malaria. For some CHWs, access to malaria Ag-RDTs facilitated offering and accepting the COVID-19 test during consultation.

However, CHWs are aware that patients may find it challenging to refuse the CHWs’ proposal, raising questions about patients’ ability to express their consent in this context. Some patients negotiate by saying, “I’ll do it later, let’s start with malaria treatment,” or refuse outright with responses like, “I didn’t come for that,” or “the chief doesn’t want it.”

### Cost-effectiveness component.

In the intervention arm, CHWs reached 1,539 patients and COVID-19 Ag-RDTs testing was carried out for 388 patients, resulting in 252 Ag-RDTs per 1,000 patients. In the SOC arm, CHWs reached 1,016 patients, of whom 183 were referred to CHCs for COVID-19 Ag-RDTs testing, with only 10 undergoing an Ag-RDT, resulting in 9.8 Ag-RDTs per 1,000 patients.

The total intervention cost was 16,727 USD compared to 14,492 USD for the SOC, with human resources representing the main cost (Figure 2). The average cost per COVID-19 Ag-RDT test was 43.1 USD in the intervention and 1,449.2 USD in the SOC.

The estimated incremental cost-effectiveness ratio (ICER) showed that each additional COVID-19 Ag-RDT test performed at the community level by CHWs costs on average 9.2 USD representing 12% of Mali’s monthly GDP, per capita.

## DISCUSSION

This study demonstrates that CHWs can follow testing protocol accurately and interpret COVID-19 Ag-RDTs results correctly. This finding aligns with existing evidence that CHWs are proficient in performing RDTs, notably for malaria^10^ and also HIV diagnosis.^11^ Acquiring this new skill boosts CHWs’ satisfaction, as shown with malaria Ag-RDT^12^ and helps solidify their role in the community and with their supervisors. Community trust in CHWs, along with community leader involvement and awareness-raising activities, contributed to the study’s success, with the availability of free and rapid Ag-RDTs enhancing participation in intervention areas. While the intervention was implemented on a limited scale and duration, a comparison with the national strategy highlights the positive impact of decentralising COVID-19 diagnostics to the community level.

This study highlights the potential of integrating malaria and COVID-19 diagnostics, particularly during peak malaria transmission seasons. Since the symptoms of COVID-19 and malaria can overlap significantly, it is often challenging to distinguish between the two based on clinical suspicion alone: 89% of febrile patients had diagnostic criteria for COVID-19, while 90% of suspected COVID-19 patients also presented with fever, triggering malaria suspicion.

An unexpected finding was the lower access to malaria screening and treatment in the SOC arm, even though these services are routinely available in the community. This lower access may be attributed to national recommendation advising that during the COVID-19 pandemic, the initial step in patient management should involve COVID-19 clinical screening. As a result, patients meeting COVID-19 screening/testing criteria were referred to health centers, which may have inadvertently limited access to malaria screening and treatment during the peak transmission season. The established trust between CHWs and their communities, which helped facilitate COVID-19 test acceptance, also proved limiting, as some patients hesitated to tell CHWs they wouldn’t go to the health center for further care.

One of the study’s key findings is that many patients refuse to go to healthcare facilities, even after agreeing to be referred, because of transport costs and time constraints, but also because they feel their symptoms are not severe enough to warrant the trip to health facilities. These issues have already been documented in Mali for child and women’s health: distance from HCF, household income, level of education, and men’s decision-making power are the main factors associated with low attendance to HCF.^13^^,^^14^

Although deploying CHWs in Mali helps address these challenge by decentralizing primary healthcare services for individuals residing more than 5km from the health facilities, our study suggests a need to provide, whenever possible, a full package of medical services, including not only diagnostic, but also severity assessment, to on-site treatment of mild diseases, similar to malaria services.

This study is limited by its geographical scope and short duration, which may constrain the generalizability of its findings. The low prevalence of COVID-19 during the study period limited our ability to assess the access to care for patients who tested positive in the community and the feasibility of community-based testing strategies on COVID-19 contacts. This low prevalence also did not allow us to perform a cost-effectiveness analysis based on COVID-19 detection. Instead, we assessed the efficacy of performing a test, being aware that the value of a negative result in terms of public health is more than limited in the absence of broader interventions.

The study’s timing during the rainy season may have boosted recruitment due to the high malaria incidence while increasing referral refusals due to agricultural activity during this period. Additionally, CHWs’ incentivization during the study, on-site and remote supervision, and provision of tests, sampling and protection materials, may have overestimated the strategy’s effectiveness, as these additional activities may not be sustainable.

## CONCLUSION

This study confirms the central role of CHWs in healthcare decentralization in Mali, supported by community trust and the challenges associated with limited access to HCFs. Providing of COVID-19 Ag-RDTs at the CHW level, along with initial training and supervision is an effective and well accepted approach.

Integrating Ag-RDTs for emerging pandemics like COVID-19 with those for prevalent diseases like malaria offers a straightforward method to enhance diagnosis and surveillance at the community level. However, even after a diagnosis is made in the community, barriers to accessing health centers remain, reinforcing the importance of offering care at the community level for non-severe cases, when feasible.

Lastly, the sustainable funding and availability of CHWs are ongoing challenges in Mali, underscoring the need for careful planning when expanding CHWs’ package of care and its consequences in terms of workload in alignment with national health priorities.
